# Synthesis of Highly Expandable Poly(methacrylimide) (PMI) Precursor Beads Through Optimized Suspension Polymerization of MAA-MAN-tBMA Copolymers

**DOI:** 10.3390/polym17010089

**Published:** 2024-12-31

**Authors:** Haozhe Wang, Yusong Gao, Zhiying Yin, Jianbin Qin, Yongsheng Zhao, Guangcheng Zhang

**Affiliations:** School of Chemistry and Chemical Engineering, Northwestern Polytechnical University, Xi’an 710072, China

**Keywords:** aqueous-phase suspension polymerization, salting-out technique, bead size regulation, PMI foam beads, thermal-induced free foaming

## Abstract

Bead-foaming technology effectively addresses production cycles, polymerization control, and cellular structure defects in conventional bulk foaming, especially in high-performance PMI foams. In this work, highly expandable PMI beads were synthesized based on the aqueous suspension polymerization of methacrylic acid-methacrylonitrile-tert-butyl methacrylate (MAA-MAN-tBMA) copolymers. The suspension polymerization was stabilized by reducing the solubility of MAA by the salting-out effect and replacing formamide (a common PMI foaming agent) with tBMA. The polymerization process was optimized by varying salting-out agents, dispersants, water-to-oil ratio (WOR), and stirring speed to achieve uniform bead sizes (0.2–0.4 mm) and high bead yields (>70%). The expansion ratio of the beads can be easily tuned by adjusting tBMA content and foaming time and temperature. Beads with 10%tBMA can reach up to 64 times under a free-forming process at 240 °C, which serves as an excellent precursor toward high-performance in-mold foaming PMI. The beads exhibit excellent in-mold foaming capabilities, thermal stability (Td = 392 °C), and mechanical properties. This work provides a technical foundation for the bead-foaming technology of PMI foams, reducing the cost of PMI foam production and providing the possibility to expand the application of PMI foam in civilian use.

## 1. Introduction

Poly(methacrylimide) (PMI) foam is a high-performance, thermosetting rigid foam material with a nearly 100% closed-cell foam structure, offering excellent strength, stiffness, and heat resistance compared to rigid structural foams with similar density [1]. PMI foam is widely used as a foam core of the sandwich structure in aerospace and automotive manufacturing due to its excellent compressive and thermal resistance [2,3]. Additionally, its superior sound and thermal insulation properties make it suitable for high-speed train fairings and high-end audio casings, while its transparency to electromagnetic radiation allows for applications in MRI systems and radar radomes [4,5].

PMI foam originated from a patent published by Sekisui Chemical in Japan in 1962 [4], in which 2-methacrylic acid (MAA) and 2-methacrylamide (MAM) were utilized as comonomers. Through radical polymerization, an expandable plate-like polymer was produced, which forms six-membered imide ring structures during the thermal foaming process. Although this product demonstrated excellent heat and corrosion resistance, shrinkage occurred with prolonged exposure to water or humid air. In 1968, improvements in water resistance were introduced by Röhm in Germany, who incorporated methyl acrylonitrile (MAN) as a copolymer monomer [5]. Currently, mainstream PMI foam production is based on the use of MAN and MAA as primary monomers, combined with free radical initiators and physical foaming agents. An expandable precursor copolymer plate was produced by bulk polymerization in a 35–60 °C water bath, then subjected to high-temperature foaming and post-processing to form foam boards. Nevertheless, the long reaction cycle and the risk of uncontrolled polymerization in thicker boards limit its development. Furthermore, since PMI cannot be processed by melting, methods such as CNC machining are required to cut foams into complex structures for different requirements. This results in low efficiency and high material loss, further limiting its widespread application.

To enable the rapid and low material loss shaping of PMI products, a patented method was developed by Evonik Degussa GmbH, in which bulk-polymerized PMI plates are broken into particles and foamed within a mold with the required shape [6]. However, this progress still required an extra operation, and these particles exhibited uncontrolled shape and size, leading to poor interfacial adhesion and diminished performance relative to integrally foamed products, even when surface adhesives were applied. To solve these problems, the central challenge of our study is to develop an efficient approach for synthesizing uniform and highly expandable PMI precursor beads using suspension polymerization, overcoming the limitations of existing techniques in terms of size uniformity and operational costs.

Suspension polymerization, a well-established technique, involves the dispersion of water-insoluble monomers into stabilized droplets, with vigorous stirring producing polymer beads [7]. Typically, a soluble initiator is employed in the liquid monomer phase. In cases where partially or fully water-soluble monomers are used, some monomers dissolve in the aqueous phase. For fully water-soluble monomers, suspended micro-droplets may fail to form, potentially leading to emulsification or polymer precipitation, which can result in poor hydraulic properties and clogging of equipment due to bead agglomeration. Zhou et al. [8] utilized suspension polymerization to prepare PMI expandable precursors but obtained a viscoelastic prepolymer rather than beads, which was subsequently processed into expandable sheets by rolling. Zhang et al. [9] synthesized expandable acrylonitrile (AN) type PMI precursor beads with diameters of 1–2 mm through the suspension polymerization of AN and MAA. However, a significant reactivity difference between AN and MAA resulted in lower imide cyclization efficiency compared to MAA-MAN copolymers, and the resulting beads were relatively large [10].

**Scheme 1:**
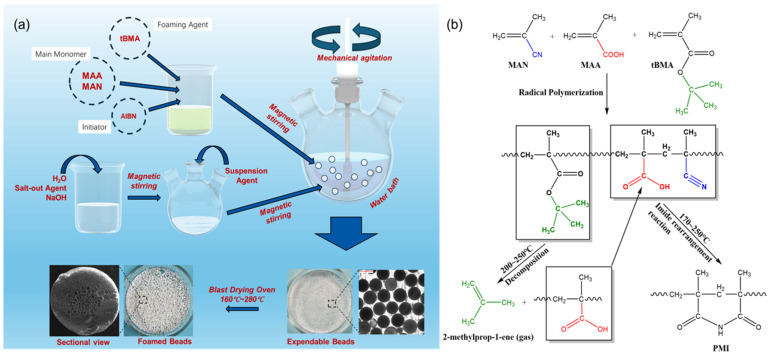
(**a**) Schematic illustration of the suspension polymerization and foaming process for expandable MAA-MAN-tBMA copolymer beads, along with images of the foamed products; (**b**) Mechanism of MAA-MAN-tBMA copolymerization and thermal foaming.

This study used multiple optimization strategies to overcome the challenges of synthesizing PMI precursor beads through aqueous suspension polymerization. The salting-out agent was applied to reduce the solubility of MAA, water-soluble foaming agent formamide was substituted with comonomer tBMA, and careful adjustments to factors such as dispersant and concentration, stirring speed, WOR (water-to-oil ratio) optimized the polymerization process. As a result, PMI precursor beads with a narrow size distribution (0.1–1.0 mm), a high yield (over 70%), and a remarkable free expansion ratio of up to 64-fold were successfully synthesized. The synthesis process and primary chemical reactions are illustrated in Scheme 1.

The advances presented in this study not only address technical limitations in PMI bead foaming but also have the potential to expand the application of PMI in industries such as consumer-grade automotive and electronics, due to the flexibility and control offered by the optimized process, which simplifies production and improves efficiency.

## 2. Materials and Methods

### 2.1. Materials

Methyl acrylonitrile (MAN, Analytical Reagent (AR)) was obtained from Hunan Huateng Pharmaceutical Co., Ltd. (Changsha, China) Methyl methacrylate (MAA, AR), azobisisobutyronitrile (AR, AIBN), tert-butyl methacrylate (tBMA, AR), and poly(methacrylic acid) sodium salt (PMAA-Na, AR) were sourced from Aladdin Biochemical Technology Co., Ltd. (Shanghai, China). Deionized (DI) water was prepared in the laboratory using a high-purity water purification system. Sodium chloride (NaCl, AR), potassium chloride (KCl, AR), sodium sulfate (Na_2_SO_4_, AR), potassium sulfate (K_2_SO_4_, AR), sodium hydroxide (NaOH, AR), polyvinyl alcohol-1799 (PVA-1799, alcoholysis 98~99% (mol/mol)), and sodium poly(methacrylate) (PMMA-Na, Mw = 4000–6000, 40 wt.% in H_2_O) were provided by Macklin Biochemical Technology Co., Ltd. (Shanghai, China). Polyvinyl alcohol-124(PVA-124, alcoholysis 98~99% (mol/mol)) was procured from Guanghua Sci-Tech Co., Ltd. (Shantou, Guangdong, China). Polymerization temperature was controlled by the oil pot (DF-101T, XIUILAB, Shanghai, China, and string speed was controlled by the digital stirring apparatus (OS20-S, DRAGONLAB, Beijing, China). The microstructure of the beads and foam was observed using a scanning electron microscope (TESCANVEGA3LMH VEGA 3 LMH, Brno–Kohoutovice, Czech Republic) and microscope (DMM-300C, Caikon, Shanghai, China). The viscosity information during the polymerization process was measured using a viscometer (CAP2000+, Brookfield, Middleboro, MA, USA).

### 2.2. Suspension Polymerization and Free Foaming of Expandable MAA-MAN-tBMA Beads

#### 2.2.1. Aqueous Phase Preparation

A measured quantity of salting-out agent (5–20% mass of aqueous phase), 0.016 g NaOH, and 100 mL of deionized (DI) water was combined in a 500 mL three-neck flask with magnetic stirring. A specific volume of dispersant solution was added into the flask and stirred continuously until a homogeneous mixture was achieved.

#### 2.2.2. Organic Phase Preparation

A measured quantity of MAN (10.1 g), MAA (12.9 g), tBMA (0–15% mass of organic phase), and AIBN (0.2% mass of organic phase) was weighed with an analytical balance before being mixed in a beaker and then transferred to a constant pressure separating funnel.

#### 2.2.3. Suspension Polymerization Process

The reaction setup was maintained at 80 °C in an oil bath, with agitation provided by an 85 mm polytetrafluoroethylene stirring paddle. Nitrogen gas was introduced at a flow rate of 25 mL/min for 20 min to displace residual oxygen. The oil phase was introduced into the aqueous phase through the constant pressure separating funnel in 2 min. Consistent shear force from the stirring paddle maintained uniform droplet suspension throughout the process. Samples gained from different controlled experiments were listed in supporting information Table S1.

#### 2.2.4. Post-Treatment Process

The resulting product beads were filtered through 80–100 meshes and then transferred to a 1 L beaker containing 500 mL of cold water. The beads were stirred and rinsed multiple times to remove any residual salting-out agents, dispersants, and unreacted monomers. Surface moisture was removed by vacuum filtration to minimize adhesion between beads during drying. Finally, the beads were placed in a forced-air oven at 110 °C for 2 h, stirring every 20 min to prevent surface adhesion.

#### 2.2.5. Free Foaming of Beads

The dried beads were placed in a forced-air oven at 230 °C for free thermal expansion. A large container was preferred to provide ample space for expansion, minimizing the risk of deformation, surface adhesion, or other factors that could compromise the expansion ratio due to bead compression.

### 2.3. Characterization

#### 2.3.1. Monomer Solubility Test

To test monomer solubility, an oil phase with an MAA molar ratio of 1:1 was prepared and combined with an aqueous phase containing a salting-out agent with a WOR of 4:1. Following MAA redistribution between two phases. The mixture was allowed to settle for phase separation. Using a pear-shaped separatory funnel, the oil and aqueous phases were separated. The MAA solubility (kg/L) was determined by a titration experiment with 0.1 M NaOH(aq). 

#### 2.3.2. Measurement of WOR

WOR is defined according to Equation (1):

(1) $$WOR=\frac{m_{water}}{m_{oil}}$$

where *m_water_* represents the mass of the aqueous phase, and *m_oil_* is the mass of the organic phase.

#### 2.3.3. Measurement of Bead Size

The diameters of N beads (where N ≥ 100) were measured using ImageJ software (version 1.53k), which was calibrated using the built-in scale of the microscope, according to optical microscope photographs. The bead size-frequency distributions and cumulative frequency distribution curves were calculated. Since the particle size distribution results approximated a normal distribution, D50 was used to represent the intuitive average bead size (particle sizes at 50%). The bead size span was employed to characterize the bead size distribution. The calculation formula is as follows:

(2) $$Span=\frac{D_{90}-D_{10}}{D_{50}}$$

The formula *D_1_*_0_ and *D_90_* represent the particle sizes at 10% and 90% cumulative distribution, respectively, which were recorded in Table S2.

#### 2.3.4. Measurement of Bead Expansion Ratio

The expansion ratio R was determined by comparing the volumes of beads before and after free thermal expansion. Since the beads retained a nearly spherical shape throughout the expansion, the expansion ratio was calculated as the average volume ratio of each bead before and after expansion according to Equation (3):

(3) $$Expansion\;Ratio=\frac{1}{N}\sum {\left(\frac{Dᵢ}{dᵢ}\right)}^{3}$$

where *d_i_* (μm) is the diameter of the bead before expansion. *D_i_* (μm) represents the diameter of the bead after expansion. *N* (≥100) is the number of beads measured after expansion

#### 2.3.5. Measurement of Bead Yield

Bead yield P was calculated as follows:

(4) $$Yield=\frac{m_{1}}{m_{0}}\times 100\%$$

where *m_1_* (g) is the mass of beads obtained after sieving and *m_0_* (g) is the initial mass of the monomer feed.

#### 2.3.6. Measurement of Comonomer Conversion

At specified reaction intervals, a syringe is used to withdraw an aliquot of the reaction mixture. After allowing the sample to settle and phase-separate, the upper organic phase is collected and weighed, denoted as *m*_0_ (g). The sample is then dried at 140 °C to evaporate unreacted monomers, as the polymerized macromolecules remain stable and do not volatilize at this temperature. The mass of the residual solid represents the polymerized comonomer content, denoted as *m_p_* (g). Comonomer conversion was calculated as follows:

(5) $$Conversion=\frac{m_{p}}{m_{0}}\times 100\%$$

## 3. Results and Discussion

### 3.1. Effect of Salting-Out Agent of PMI Precursor Beads

During aqueous-phase suspension polymerization, MAA, along with a small portion of AIBN, tends to dissolve into the aqueous phase due to its high solubility (saturated concentration of 89 × 10^−3^ kg/L [11]). The monomer in aqueous phase destabilizes the suspension, causing agglomeration and reducing both bead yield and PMI precursor bead quality [12].

**Figure 1:**
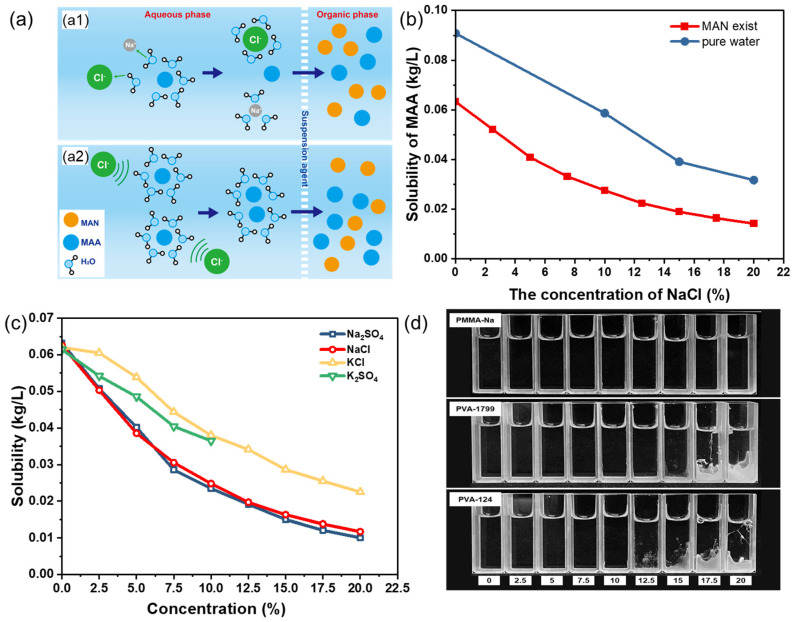
(**a**) Schematic illustration of the effect of salting-out ions on water-soluble molecules; Ions lead to precipitation by (**a1**) Ions capture H_2_O and (**a2**) MAA aggregation (**b**) Effect of MAN presence on the solubility of MAA in NaCl aqueous solution; (**c**) Effect of type and concentration of salting-out agent on MAA solubility; (**d**) Effect of NaCl concentration on the dispersants’ solubility.

To solve this problem, strong electrolytes, as salting-out agents, were introduced to the aqueous phase to reduce the solubility of MAA [13,14]. Figure 1a shows the progress of salting out of MAA. The existence of salting-out agents reduces the binding of water molecules to MAA (Figure 1(a1)), and high-charge-density anions promote MAA aggregation through electrostatic repulsion and hydrophobic effect (Figure 1(a2)) [15]. Moreover, our previous work [9] has produced AN-MAA copolymer beads successfully by using 20% NaCl (aq) as an aqueous phase in an AN-MAA suspension polymerization system, which demonstrates the feasibility of decreasing the solubility of MAA by introducing Na+ and Cl^−^. Additionally, the highly polar nitrile group in MAN within the oil phase forms strong interactions with MAA, further enhancing the salting-out effect. As shown in Figure 1b, with the existence of MAN, the solubility of MAA decreases by 2–4 × 10^−4^ kg/L under the same conditions. The highly polar nitrile group in MAN will form hydrogen bonds with the carboxyl group of MAA [16]. Additionally, the solubility of MAN in water is much lower than that of MAA, and this property endows an extraction effect on MAA [17], further enhancing the salting-out effect, which increases the feasibility. 

The presence of salt will inevitably lead to contamination of the product. Therefore, the salt content needs to be minimized as much as possible. It is necessary to identify a suitable salt that can significantly reduce the solubility of MAA at lower dosages. Four different electrolytes including NaCl, Na_2_SO_4_, KCl, and K_2_SO_4_ were compared based on the MAA aqueous solubility and acid-base titration method in the presence of MAN. Figure 1c presents the solubility of MAA with different salting-out agent concentrations. It was found that NaCl and Na_2_SO_4_ exhibit significantly stronger salting-out effect than that of KCl and K_2_SO_4_. The salting-out effect of NaCl and Na_2_SO_4_ on MAA aqueous solubility are comparable and the salting-out effect of Na_2_SO_4_ is slightly stronger than NaCl when the concentration is larger than 15%. Considering cost and dissolution rate, NaCl was chosen as the salting-out agent in this research.

The salting-out effect not only influences water-soluble monomers but also impacts polymer dispersing agents. As the salting-out agent concentration increases, dispersants precipitate, resulting in reduced suspension stability. To visualize the influence, solubility tests for three dispersants, including PMMA-Na, PVA-1799, and PVA-124 in NaCl solutions, were conducted at 80 °C to determine the optimal NaCl concentration. Figure 1d demonstrates that PVA-based dispersants are highly susceptible to the salting-out effect. Turbidity in PVA-1799 occurs at 12.5% NaCl, with significant precipitation at 17.5%. For PVA-124, slight precipitation begins at 12.5%, becoming more pronounced at 15%. In contrast, PMAA-Na remains clear and stable below 20% NaCl. The results clearly show that the dispersant is affected by high concentrations of the salting-out agent. The ions form hydrated complexes with water, thereby reducing hydration and, consequently, decreasing the solubility of the polymer dispersants [18].

**Figure 2:**
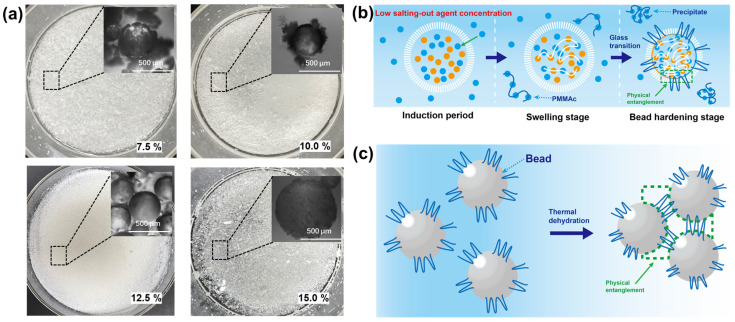
(**a**) Formation of flocs at different NaCl concentrations; (**b**) Schematic illustration of the mechanism of floc formation at low salt concentrations; (**c**) Schematic illustration of bead clumping during drying in beads produced at low salt concentrations.

A controlled experiment was conducted to evaluate the effect of NaCl concentration (5–20% in Table S1 Sample 1–7) on PMI precursor beads during suspension polymerization, with 0.4% PVA-124 as the dispersant. At low NaCl concentrations (under 10%), high MAA solubility (>2 × 10^−2^ kg/L) promotes the polymerization of hydrophilic poly(methacrylic acid (PMAAc). As is shown in Figure 2a, massive white flocculent settlement can be observed in the product photo, due to the aggregation of PMAAc chain. Some flocculent sticks to the bead surface (Figure 2b), which can be almost unremovable in post-treatment. During the drying process, dehydration of the swollen PMAAc chains on the surface causes entanglement, leading to bead agglomeration (Figure 2c). This agglomeration effect is more intense at lower NaCl concentrations. At 7.5% NaCl, approximately 6% of the MAA monomer dissolves in the aqueous phase, resulting in notable PMAAc flocs. When the NaCl concentration was increased to 12.5%, it nearly eliminated visible flocculent on the bead surface (Figure 2a), and residual PMAAc did not cause bead agglomeration during drying. When we increase the salt concentration, the effect on the dispersant becomes significant. At 15% NaCl, dispersant precipitation was observed during preparation, leading to a reduction in dispersant effectiveness, which resulted in an increase in bead size. Furthermore, an increase to 17.5% NaCl caused extensive PVA precipitation, affecting the water-oil interfacial tension and resulting in product agglomeration. Based on bead size distribution and ease of post-treatment, 12.5% NaCl was identified as the optimal concentration for the aqueous phase.

### 3.2. Effect of Dispersant on Bead Size and Distribution of PMI Precursor Beads

A dispersant is an essential part of suspension polymerization [19]. It keeps the droplets stable and dispersed through interactions with the surfaces of the monomer and polymer particles, avoiding their agglomeration and precipitation [20]. By optimizing the type and concentration of the dispersant, it is possible to regulate the dispersibility and size of the beads, which in turn affects the properties of the final product. In this study, selecting an appropriate dispersant requires consideration of the salting-out effect. The effectiveness of dispersants in concentrated electrolytes is significantly reduced due to the shielding of electrostatic repulsion, which poses substantial challenges in producing PMI precursor beads with small sizes and narrow size distributions. Therefore, a dispersant specifically suited for MAA-MAN-tBMA suspension polymerization is urgently required.

**Figure 3:**
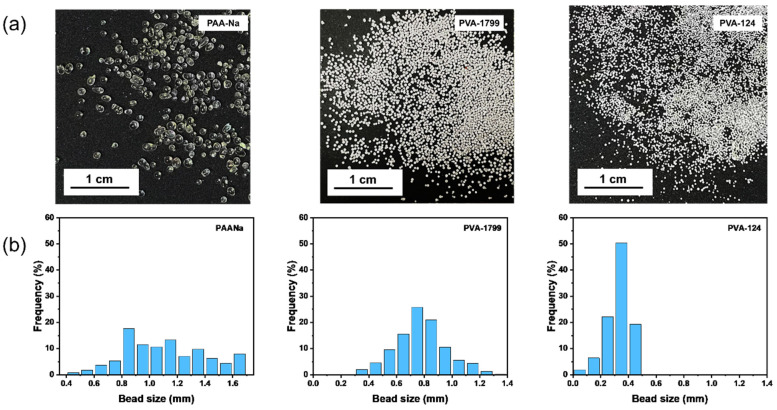
Effect of dispersant types on (**a**) appearance and (**b**) bead size distribution of beads.

To meet these requirements, controlled experiments were conducted using PVA-124, PVA-1799, and PMAA-Na to evaluate their effectiveness as dispersants [21,22]. The tests were conducted under fixed conditions as detailed in Table S1, Samples 8–10. Significant differences in bead size and distribution were observed, as shown in Figure 3. Among the dispersants tested, PMAA-Na exhibits no turbidity or precipitation in high-concentration NaCl solutions (Figure 1d), resulting in larger bead sizes, and broader size distribution with irregular shapes (Figure 3a). The irregularity of beads arises from the anionic nature of PMAA-Na, which stabilizes beads via electrostatic repulsion from –COO^−^ [23]. However, according to Lopez’s report [24], the presence of NaCl shields these interactions, diminishing its effectiveness. A notable advantage of PMAA-Na is the high transparency of the resulting beads, likely due to its compositional compatibility with the copolymer, which minimizes phase separation. PVA-based dispersants led to higher bead yields and enhanced sphericity, although they did precipitate at higher concentrations of NaCl. PVA-124 showed superior dispersing ability, producing smaller beads with a narrower size distribution.

In conclusion, based on bead size, distribution, yield, and sphericity in Table 1, PVA dispersants outperformed in this system, and the higher molecular weight of PVA-124 compared to PVA-1799 enhances its dispersing capability at the same concentration, making it the preferred dispersant in this study. However, PVA-1799, with its lower molecular weight, also has practical applications. Due to the limited solubility of PVA-based dispersants in water, they must be prepared as aqueous solutions before suspension polymerization. PVA-1799 offers higher solubility and lower viscosity in solutions than PVA-124, which means it can be removed easily in post-treatment. Therefore, PVA-1799 serves as a viable alternative to produce beads larger than 0.5 mm.

**Figure 4:**
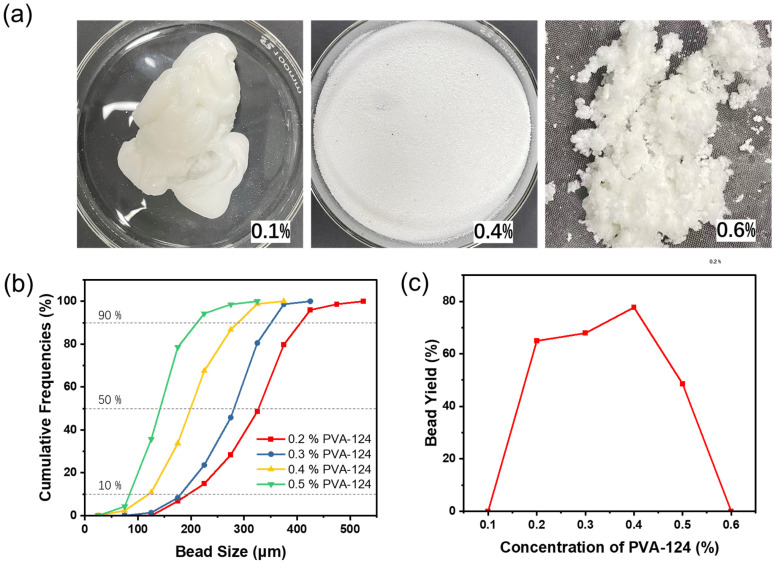
(**a**) Formation of aggregation and clumps at different PVA-124 concentrations; (**b**) Effect of PVA-124 concentration on bead size distribution; (**c**) Effect of dispersant concentration on bead yield.

The effects of PVA-124 concentrations on the product under identical conditions are shown in Figure 4a. At concentrations of 0.1% and 0.6% (by mass of water), varying degrees of agglomeration were observed during suspension polymerization. At low dispersant concentrations (0.1%), the dispersion capability is insufficient to prevent bead coalescence at a stirring speed of 140 rpm, as the shear force generated is not strong enough. Increasing the stirring speed could help, but adjustments to the formulation at higher speeds have a minimal impact on bead quality, making it difficult to produce beads that meet various specifications. A high dispersant concentration (0.6%) leads to precipitation in the salt solution. This results in bead bridging and clumping during polymerization. Although these clumps can be broken down into smaller beads after drying, the process is cumbersome. Excess dispersant also tends to remain on the product surface [25], requiring additional washing and purification steps that complicate post-treatment [26]. Figure 4b shows the statistical analysis of bead size and distribution at different dispersant concentrations. For every 0.1% increase in dispersant concentration, bead size diminishes by approximately 50 μm. This trend is evident only within the 0.2–0.5% concentration range, indicating that increased dispersant concentration within this range enhances bead dispersion. However, excessively high or low dispersant concentrations lead to agglomeration and irregular bead formation. The yield-versus-dispersant concentration curve in Figure 4c indicates that within a concentration range of 0.2–0.4%, bead yield remains consistent, with no signs of beads clumping in the product. However, when the dispersant concentration exceeds 0.5%, a noticeable decline in yield occurs due to clumping, likely related to the limited solubility of PVA-124 in a 12.5% NaCl solution. In this state, a small number of PVA-124 precipitates out of the system, entrapping some beads and forming hard aggregates, thereby reducing yield.

Table 2 lists the effect of PVA-124 concentration on suspension polymerization, and 0.4% PVA-124 was selected to achieve a uniform bead size distribution and high bead yield with minimal dispersant usage.

### 3.3. Effect of tBMA on Bead Yield and Expansion Ratio of PMI Precursor Beads

**Scheme 2:**
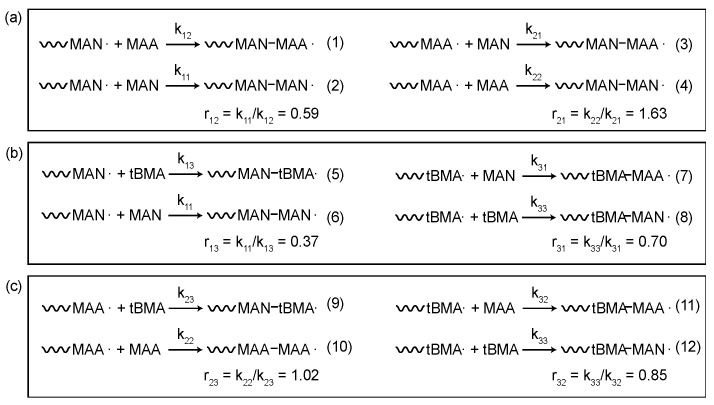
Copolymerization of MAA-MAN-tBMA and Reactivity Rations. (**a**) Competing Reactions of MAA and MAN; (**b**) Competing Reactions of MAN and tBMA; (**c**) Competing Reactions of MAA and tBMA.

Traditional PMI precursors commonly employ physical foaming agents, for example, formamide [1,26], which do not participate in polymerization and thus exhibit poor dispersion stability. Furthermore, its water solubility makes it unsuitable for aqueous suspension polymerization. tBMA significantly enhances stability and distribution in precursor beads, according to our previous research, and provides harmless gas to meet the requirement of environmental protection. Moreover, the introduction of tBMA also changes the consistency of PMI precursor polymer chains. In the copolymerization of MAA-MAN, as shown by the reactivity ratios in Scheme 2a, MAN radicals tend to react with MAA, yet MAA has a higher propensity for self-polymerization. A related study [27] indicates that with an initial MAN/MAA feed ratio of 1.514, the copolymer product has a monomer incorporation ratio of only 0.94, suggesting that MAA self-polymerizes extensively, limiting copolymerization of MAN into the polymer chain and impacting yield as well as subsequent imidization reactions. The addition of tBMA modifies this behavior, as reactions illustrated in Scheme 2b,c demonstrate. According to Chen’s report [28], tBMA and MAN tend to alternate copolymerization, facilitating an even distribution of tBMA along the chain. Although the self-polymerization rate constant of MAA (k_22_) slightly exceeds the rate (k_23_) at which MAA radicals combine with tBMA, the difference is minimal (r_23_ = 1.02). Consequently, the inclusion of tBMA increases MAN incorporation in the copolymer, thereby enhancing yield.

**Figure 5:**
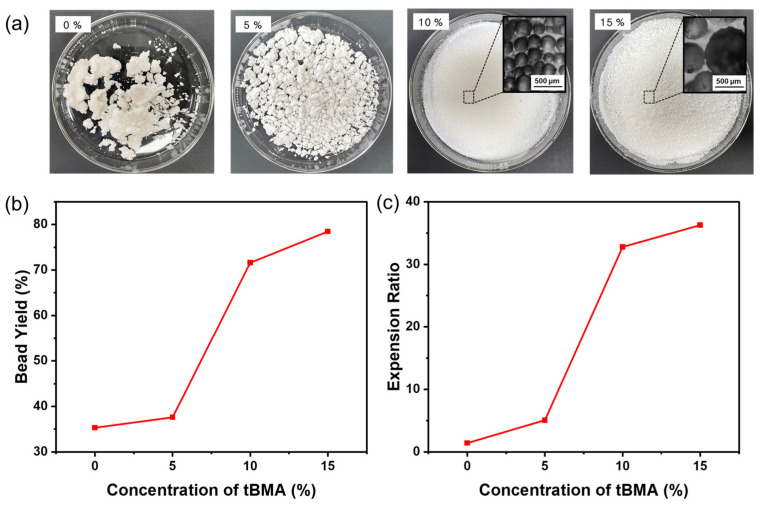
(**a**) Formation of clumps at different tBMA initial concentrations; (**b**) Effect of tBMA initial concentration on bead yield. (**c**) Effect of initial tBMA concentration on bead expansion.

To examine the effects of tBMA content on beads in suspension polymerization, tBMA was added to the oil phase at concentrations ranging from 0% to 15% (by mass of the organic phase) (Table S1 Sample 29–32). Figure 5 shows that tBMA content significantly affects clumping during polymerization. Although clumps can be dispersed into beads with shearing force, it has a negative impact on bead yield. Clumping primarily results from the surface enrichment of hydrophilic monomers, which promotes chain entanglement. This outcome is associated with the hydrophobicity of tBMA due to its large, non-polar tert-butyl group, which outweighs the minor polarity of its ester group. This characteristic reduces clumping and improves the bead yield.

The results of the experiment prove that when tBMA is lower than 5%, system stability declines, resulting in significant clumping and a yield below 50%. When the concentration of tBMA was increased to 10%, it significantly enhanced system stability and increased the yield to 71.6%. When the concentration was kept at a higher level, the yield increased to a new level, but the bead turned out to be larger, which was not our expectation.

A preliminary investigation was conducted to examine the influence of initial tBMA concentration on the expansion ratio. Samples with varying tBMA contents were tested under free-foaming conditions at 230 °C, and the comparison before and after expansion and the particle size distribution are shown in Figure S1. Relationship between tBMA concentration and expansion ratio is shown in Figure 5c, which demonstrates that tBMA presence is one of the critical factors for bead expansion. Beads without tBMA showed negligible expansion (1.4 times). At an initial tBMA concentration of 5%, thermal expansion was detected, though it was low (5.07 times), and did not reach an expected level. A substantial enhancement in the expansion ratio was observed at 10%, reaching 32.78 times. However, further increases in tBMA concentration did not sustain this trend; at 15%, the expansion ratio rose marginally to 36.28 times. Based on a comprehensive analysis of bead yield, size, and expansion ability presented in Table 3, 10% tBMA was identified as the optimal concentration for use as the third monomer in copolymerization.

### 3.4. Effect of Water-to-Oil Ratio on Bead Yield and Bead Size of PMI Precursor Beads

WOR is one of the critical factors in determining the bead yield of the production in suspension polymerization. A lower WOR generally increases bead yield, especially for water-soluble monomers [29], as more monomer molecules remain in the oil phase. Additionally, reducing WOR can also improve equipment utilization, which is beneficial for industrial-scale production. However, an excessively low WOR increases the risk of agglomeration and clumping due to a higher probability of droplet collisions. In contrast, a high WOR stabilizes the suspension and reduces droplet collisions, resulting in smaller beads with a narrower size distribution [30].

**Figure 6:**
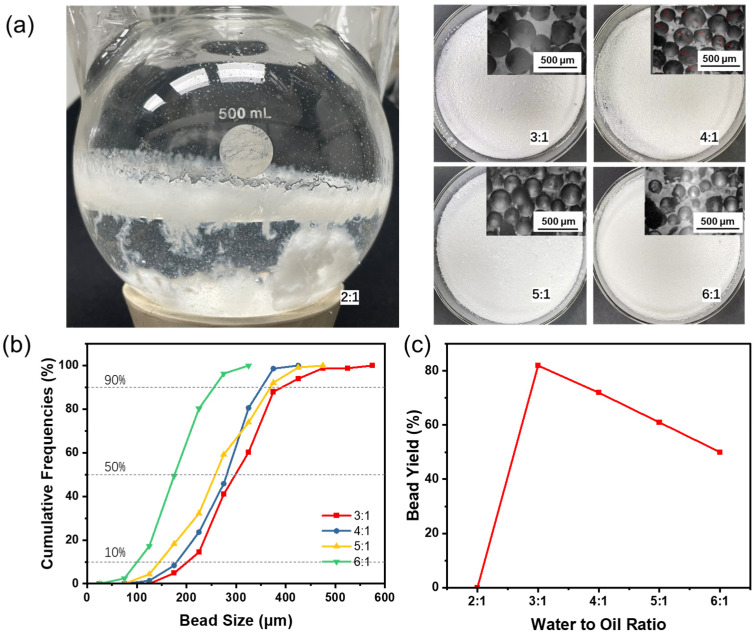
(**a**) Effect of WORs on product appearance; (**b**) Effect of WOR on bead size distribution; (**c**) Effect of WOR on bead yield.

To identify the optimal WOR, mass ratios from 2:1 to 6:1 were tested in a controlled experiment (Table S1 Sample 24–28), with yield and bead size distribution analyzed. Product images and optical micrographs are shown in Figure 6a. Agglomeration was observed at the 2:1 ratio, while beads were successfully formed at all other ratios. Figure 6b shows the bead size distribution. Bead size decreases slightly as the WOR increases from 3:1 to 5:1. This is related to the more uniform dispersion of oil-phase droplets in the aqueous phase, which also reduces the likelihood of droplet collision and agglomeration. Figure 6c shows the relationship between WOR and yield. When the WOR decreased from 6:1 to 3:1, the yield increased from 48% to 82%. This increase in yield can be attributed to the reduced dissolution of monomers in the aqueous phase, which is consistent with the findings of Lu et al. [29] regarding the limitations of conversion in aqueous suspension polymerization of water-soluble monomers. Although the yield is highest at a 3:1 ratio, the stability of the system is significantly lower compared to the 4:1 ratio. This is due to the increased probability of bead collisions at lower WOR.

Based on a comprehensive analysis of reaction stability and yield in Table 4, the experiment was conducted with a WOR of 4:1.

### 3.5. Effect of Stirring Speed on Bead Size and Span of PMI Precursor Beads

Bead size is determined by droplet breakup and aggregation [31]. Stirring-induced shear forces promote the breakup of the oil phase, while the oil phase aggregates spontaneously. With fixed phase components and WOR, bead size is controlled by adjusting the stirring speed [3,32].

**Figure 7:**
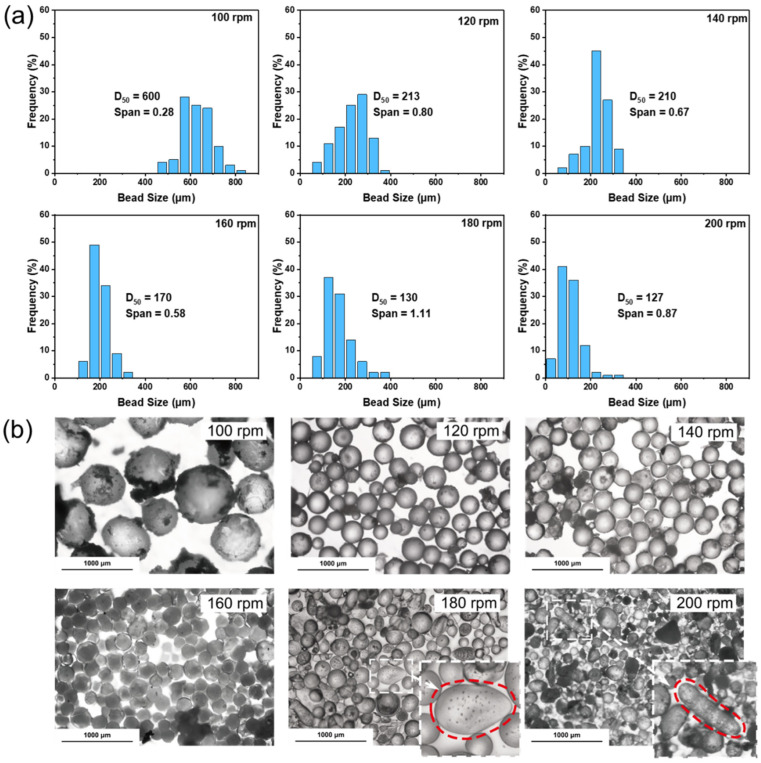
(**a**) Effect of stirring speed on bead size distribution; (**b**) Effect of stirring speed on beads’ microscopic morphology.

This study investigates the effect of stirring speed on bead size. Under optimal conditions (Table S1 Sample 17–23), stirring speeds from 100 rpm to 200 rpm were tested. Bead size distributions at each speed are shown in Figure 7a. Increasing stirring speed gradually decreases bead size in the range of 100–160 rpm because high stirring speeds and shear forces reduce coalescence of droplets [33]. At speeds above 180 rpm, the average bead size continues to decrease, however, the size distribution widens. This widening may result from intense shear forces deforming beads in the viscoelastic state, which reduces sphericity. As shown in Figure 7b, elliptical and rod-shaped beads appear at speeds exceeding 180 rpm, and stronger turbulence increases collision frequency among beads, leading to the formation of larger beads within the product [34], which is undesirable in production.

The result suggests that using PVA-124 as the dispersant, bead size can be adjusted between 100–800 μm by varying the stirring speed to meet production requirements. To produce beads smaller than 400 μm, stirring should be maintained within the 140–160 rpm range. For larger sizes, fine adjustments can be made between 100–120 rpm. Bead size remains below 1 mm (without agglomeration), even at lower stirring speeds.

### 3.6. Effect of Reaction Time on Comonomer Conversion Rate

**Figure 8:**
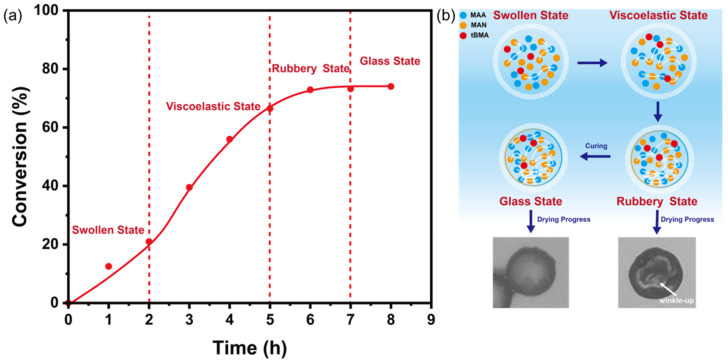
(**a**) Comonomer conversion rate over time at 80 °C; (**b**) Schematic illustration of bead structure at different polymerization stages.

The effect of reaction time on comonomer conversion at 80 °C was investigated. Comonomer conversion rate was recorded (Figure 8a). The resulting curve was analyzed and divided into sections based on the observed system state and product morphology. During the initiation phase, monomers were dispersed as droplets within the aqueous phase. As polymerization progresses, polymer formation within these droplets causes swelling, leading to a transition in the system’s appearance from clear to milky white. The slope of the conversion curve increased at a conversion rate of 20%, which resulted from the higher polymer concentration within the droplets. This increase in polymer concentration induces a viscoelastic state in the system (Table S3), triggering mild auto-acceleration. Nevertheless, efficient heat dissipation in suspension polymerization mitigates this effect, only resulting in a slight increase in the curve’s slope.

After the conversion rate reached 60%, the reaction rate decreased due to the reduced comonomer concentration. Beads began to settle, the surface viscosity declined, reducing the risk of agglomeration. Produced beads with elasticity and susceptible to wrinkle-up during drying. This issue arises as unconverted monomers within the beads escape during drying. The reaction must continue, allowing higher comonomer conversion in the curing stage. When the conversion rate reaches 70%, the beads transition to a glassy state, at which stage the reaction can be terminated. The product beads are then filtered, screened, and dried without winkle-up.

### 3.7. Effect of Foaming Time and Temperature on Bead Expansion Ratio

The decomposition temperature of the foaming agent tBMA, responsible for releasing isobutylene gas, is approximately 200 °C according to Grant’s report [35], while imide rearrangement and cyclization occur at temperatures above 160 °C [36]. To improve energy efficiency, the foaming temperature was controlled at the lowest achievable level within the experimental range of 160–280 °C, using beads containing 10% tBMA.

**Figure 9:**
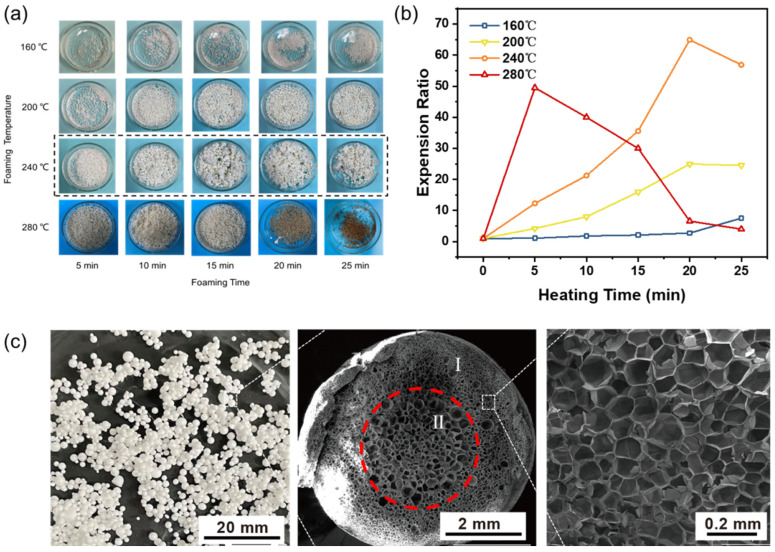
(**a**) Expansion and yellowing of beads at different foaming temperatures and times; (**b**) Effect of foaming time of expansion ratio at different foaming temperatures; (**c**) Microscopic morphology of foamed beads at 240 °C and its SEM of cross-section. Area I and II was divided according to the cell size.

Foaming temperatures of 160 °C, 200 °C, 240 °C, and 280 °C were selected to investigate the relationship between bead expansion ratio and foaming time. The same batch of beads was heated for 5, 10, 15, 20, and 25 min at each temperature, with the expanded beads shown in Figure 9a. The expansion time-temperature curve (Figure 9b) demonstrates that within the range from 160 to 240 °C, the maximum expansion ratio increases with temperature, reaching up to 56 times. At 160 °C, the beads exhibited minimal expansion, achieving only 7.53 times after 25 min of free-foaming. The expansion ratio increases with temperature, reaching 24.98 times at 200 °C and 64.93 times at 240 °C. However, at 280 °C, maximum expansion decreased by 49.5 times, reversing the trend observed at lower temperatures. At 160 °C, limited decomposition of tBMA produces insufficient gas pressure to expand the pore walls, resulting in minimal expansion even after prolonged heating. At 200 °C, although decomposition begins, the process remains slow, permitting crosslinking and cyclization of side-chain functional groups, which restricts chain mobility and limits expansion. At 240 °C, rapid tBMA decomposition increases chain mobility and facilitates larger pore formation despite concurrent crosslinking. At 280 °C, beads achieve maximum expansion within 5 min but subsequently begin to shrink. This rapid decomposition results in pore walls with insufficient crosslinking to retain gas pressure, leading to pore collapse and gas release, which ultimately reduces the expansion ratio. After 20 min at 280 °C, noticeable yellowing of the beads is observed. In conclusion, a foaming temperature of 240 °C for 20 min is identified as optimal.

After foaming, SEM images of the bead section were obtained in Figure 9c. Statistical analysis shows an average cell diameter of approximately 50 μm and a distinct closed-cell structure, which is consistent with the classical cell structure of PMI foams [37]. The cross-sectional morphology reveals a core-shell structure, with smaller pores on the outer layer and larger pores in the interior. This phenomenon is generally attributed to pressure differences during the foaming process. As the foaming agent decomposes under heat, the gas generated within the beads leads to pore formation and expansion. In the foam structure, larger pores have lower internal pressure than smaller ones, creating a pressure gradient that drives gas diffusion from smaller to larger pores, resulting in pore size variation.

Also, thermal conditions influence the bead structure as is shown in foamed bead cross-section. PMI foam exhibits excellent thermal insulation properties [38]. During pre-foaming in hot air, the outer layer of the beads expands, partially insulating the interior from heat. Higher temperatures promote increased cell density in area I, while the lower foaming temperature leads to larger pore sizes in area II [39]. This temperature difference is one factor contributing to the observed structure. After the foaming agent decomposes, gas escapes directly from the outermost layer, forming a dense skin layer.

### 3.8. In-Mold Foaming and Performance Study of PMI Precursor Beads

**Figure 10:**
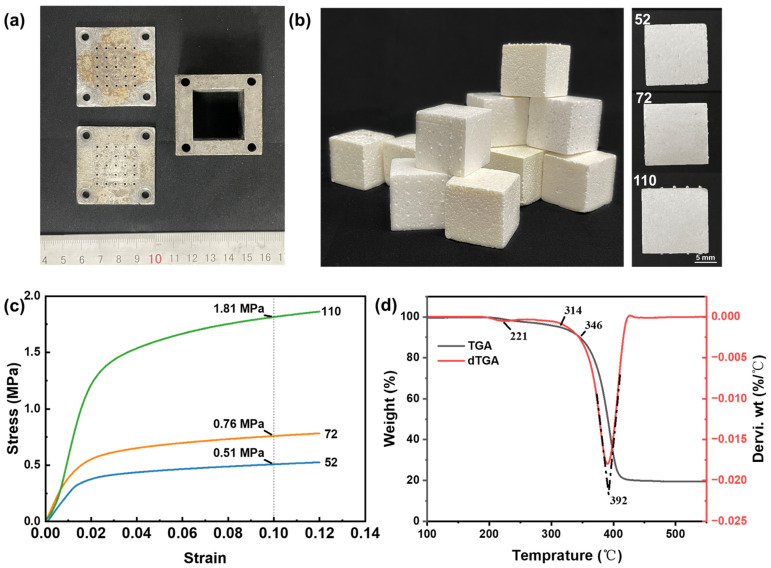
(**a**) Structure of the foaming mold; (**b**) Foaming mold and foamed PMI foam sample; (**c**) Compression stress-strain curve of foam at different densities; (**d**) TGA and DTGA curves of foam.

The feasibility of in-mold foaming of the beads was demonstrated using a 25 × 25 × 25 mm^3^ mold (as shown in Figure 10a). The in-mold foaming process was conducted at 240 °C to characterize the mechanical and thermal stability of the bead-foamed PMI foams.

The foamed samples, shown in Figure 10b, exhibited good expansion ratios across a range of densities from 53 kg/m^3^ to 110 kg/m^3^, with no noticeable voids in the mold cavity, which demonstrated its potential for producing complex structural foams. Compression tests were performed on foams of different densities using a universal testing machine. The experimental results, shown in Figure 10c, indicate that the compression stress-strain curves of the foams did not exhibit any clear yielding behavior. According to the testing standards, the stress at a strain of 0.1 was selected as the compressive strength. The compressive strength of the foam increased significantly with density, rising from 0.51 MPa at 52 kg/m^3^ to 1.81 MPa at 110 kg/m^3^. Subsequently, thermogravimetric analysis (TGA) was performed on the foams to assess their thermal stability, with the results presented in Figure 10d. The TGA curve shows that the foam undergoes a 5% weight loss at 314 °C and a 10% weight loss at 346 °C. From the DTG data, it can be inferred that the foam remains stable in terms of mass up to 221 °C, beyond which mass loss begins to occur. This weight loss is likely due to partial pyrolysis of the foam and the escape of gases caused by the rupture of the foam cells. The foam decomposition temperature (Td) was 392 °C, at which point significant degradation occurs. The bead-foamed PMI foam demonstrated heat resistance comparable to the bulk-foamed PMI that was reported by Zhang et al. [40], indicating its high heat resistance. Although the strength of this foam does not match that of bulk PMI foam [40,41], it remains impressively strong.

## 4. Conclusions

This study presents an effective approach for achieving stable PMI precursor bead formation during suspension polymerization. It was found that a 12.5% NaCl solution was used as an effective salting-out agent to reduce the solubility of MAA in water, thus promoting bead formation. When combined with 0.4% PVA-124 as a dispersant, the stability of the suspension polymerization process was further enhanced.The 10% tBMA was selected as both the foaming agent and comonomer, to achieve high yield and excellent expansion ratio simultaneously. By controlling the stirring speed (140–160 rpm) and the water-to-oil ratio (4:1), bead size, size distribution, and yield were further optimized. The optimized beads can be foamed at 240 °C for 20 min in a free-foaming process, resulting in stable bead structures and a large expansion ratio (64 times). The in-mold foam demonstrates excellent heat resistance and acceptable mechanical strength. The method for synthesizing PMI precursor beads presented in this study not only saves raw materials but also shortens processing cycles, offering promising potential for applications in aerospace, automotive, and other lightweight industries.

Despite these advances, the study’s limitations include potential impacts on product quality due to competitive monomer interactions and impurities from suspension polymerization, requiring further research.

## Figures and Tables

**Table 1:** Effects of dispersant type on PMI precursor beads.

Dispersant	D50 (μm)	Size Span	Bead Yield (%)	Sphericity
PMAA-Na	1037	0.76	≤40	Low
PVA-1799	620	0.73	≥60	High
PVA-124	171	0.69	≥60	High

**Table 2:** Effects of dispersant concentration on PMI precursor beads.

Concentration (%)	D50 (μm)	Bead Yield (%)	Product Morphology
0.1	-	-	Aggregation
0.2	327	64.92	Spherical beads
0.3	281	67.88	Spherical beads
0.4	200	77.68	Spherical beads
0.5	141	48.52	Spherical beads and clumps
0.6	-	-	Clumps

**Table 3:** Effects of initial tBMA concentration on suspension polymerization products.

Concentration of tBMA (%)	Bead Yield (%)	Morphology	Expansion Ratio
0	35.3	Beads and clump	1.40
5	37.6	Beads and clump	5.07
10	71.6	Spherical beads	32.78
15	78.48	Large beads	36.28

**Table 4:** Effects of WOR on PMI precursor beads.

Water to Oil Ratio	D50 (mm)	Bead Yield (%)	Product Morphology
2:1	-	0	Aggregation
3:1	298	82	Spherical beads
4:1	281	72	Spherical beads
5:1	258	61	Spherical beads
6:1	175	50	Spherical beads and Agglomeration

## Data Availability

The original contributions presented in this study are included in the article/Supplementary Materials. Further inquiries can be directed to the corresponding author(s).

## Supplementary Materials

The following supporting information can be downloaded at: https://www.mdpi.com/article/10.3390/polym17010089/s1, Table S1: Bead sample information; Table S2: Factors influencing bead distribution; Table S3: Viscosity data in the Polymerization Process; Figure S1: (a) The beads with different tBMA Content, and their size distribution; (b) Foamed beads with different tBMA Content, and their size distribution.
