# Supplementary material for: Synthesis of Highly Expandable Poly(methacrylimide) (PMI) Precursor Beads Through Optimized Suspension Polymerization of MAA-MAN-tBMA Copolymers
Source: Polymers (Basel). 2024 Dec 31;17(1):89. doi: 10.3390/polym17010089 (PMC11723204; doi:10.3390/polym17010089)
Supplement: Supplementary file 1 [file polymers-17-00089-s001.zip › polymers-3366699-supplementary.pdf]

Synthesis of Highly Expandable Poly(methacrylimide) (PMI) Precursor Beads Through Optimized Suspension Polymerization of MAA-MAN-tBMA Copolymers

Haozhe Wang, Yusong Gao, Zhiying Yin, Jianbin Qin, Yongsheng Zhao and Guangcheng Zhang

Supporting Infroamtion

Table S1. Bead sample information.

Sample	Salt-out agent (wt% of aquanse phase)		Dispersant (wt% of aquanse phase)		Stirring (rpm)	Water-to-oil Ratio	tBMA (wt% of oganic phase)	Reaction Time (h)
1	NaCl	5	PVA-124	0.4	140	4:1	10	8
2	NaCl	7..5	PVA-124	0.4	140	4:1	10	8
3	NaCl	10	PVA-124	0.4	140	4:1	10	8
4	NaCl	12.5	PVA-124	0.4	140	4:1	10	8
5	NaCl	15	PVA-124	0.4	140	4:1	10	8
6	NaCl	17.5	PVA-124	0.4	140	4:1	10	8
7	NaCl	20	PVA-124	0.4	140	4:1	10	8
8	NaCl	12.5	PVA-124	0.4	140	4:1	10	8
9	NaCl	12.5	PAA-Na	0.4	140	4:1	10	8
10	NaCl	12.5	PVA-1799	0.4	140	4:1	10	8
11	NaCl	12.5	PVA-124	0.1	140	4:1	10	8
12	NaCl	12.5	PVA-124	0.2	140	4:1	10	8
13	NaCl	12.5	PVA-124	0.3	140	4:1	10	8
14	NaCl	12.5	PVA-124	0.4	140	4:1	10	8
15	NaCl	12.5	PVA-124	0.5	140	4:1	10	8
16	NaCl	12.5	PVA-124	0.6	140	4:1	10	8
17	NaCl	12.5	PVA-124	0.4	80	4:1	10	8
18	NaCl	12.5	PVA-124	0.4	100	4:1	10	8
19	NaCl	12.5	PVA-124	0.4	120	4:1	10	8
20	NaCl	12.5	PVA-124	0.4	140	4:1	10	8
21	NaCl	12.5	PVA-124	0.4	160	4:1	10	8
22	NaCl	12.5	PVA-124	0.4	180	4:1	10	8
23	NaCl	12.5	PVA-124	0.4	200	4:1	10	8
24	NaCl	12.5	PVA-124	0.4	140	2:1	10	8
25	NaCl	12.5	PVA-124	0.4	140	3:1	10	8
26	NaCl	12.5	PVA-124	0.4	140	4:1	10	8
27	NaCl	12.5	PVA-124	0.4	140	5:1	10	8
28	NaCl	12.5	PVA-124	0.4	140	6:1	10	8
29	NaCl	12.5	PVA-124	0.4	140	4:1	0	8
30	NaCl	12.5	PVA-124	0.4	140	4:1	5	8
31	NaCl	12.5	PVA-124	0.4	140	4:1	10	8

32	NaCl	12.5	PVA-124	0.4	140	4:1	15	8
33	NaCl	12.5	PVA-124	0.4	140	4:1	10	1
34	NaCl	12.5	PVA-124	0.4	140	4:1	10	2
35	NaCl	12.5	PVA-124	0.4	140	4:1	10	3
36	NaCl	12.5	PVA-124	0.4	140	4:1	10	4
37	NaCl	12.5	PVA-124	0.4	140	4:1	10	5
38	NaCl	12.5	PVA-124	0.4	140	4:1	10	6
39	NaCl	12.5	PVA-124	0.4	140	4:1	10	7
40	NaCl	12.5	PVA-124	0.4	140	4:1	10	8

Highlighted portions represent single-variable controls

Table S2. Factors influencing bead distribution.

Factor		D10 (μm)	D50 (μm)	D90 (μm)	Span
WOR	3	201	298	394	0.647651
	4	179	281	351	0.6121
	5	145	258	370	0.872093
	6	100	175	255	0.885714
Dispersant Concentration （wt%）	0.2	196	327	407	0.64526
	0.3	179	281	351	0.6121
	0.4	120	200	289	0.845
	0.5	83	141	213	0.921986
String Speed (rpm)	100	527	600	695	0.28
	120	112	213	284	0.807512
	140	132	210	272	0.666667
	160	129	170	228	0.582353
	180	81	130	225	1.107692
	200	81	127	192	0.874016

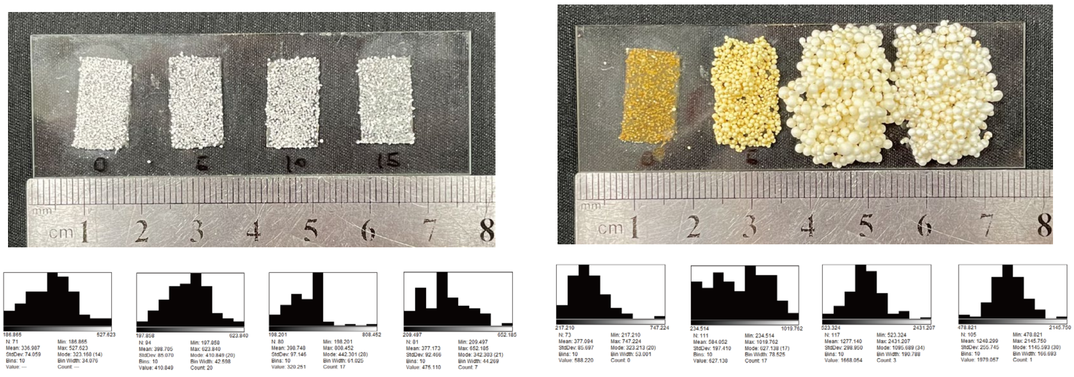

(a) (b)

Figure S1. (a) The beads with different tBMA Content, and their size distribution; (b) Foamed beads with different tBMA Content, and their size distribution.

Table S3. Viscosity data in the Polymerization Process.

Reaction time (h)	Viscosity (Poise)
1	8
2	19
4	519
6	1603
7	-[a]

[a] The sample gain in 7 hours exceeded the range of the viscometer.
